# Travel-Associated *Vibrio cholerae* O1 El Tor, Russia

**DOI:** 10.3201/eid2211.151727

**Published:** 2016-11

**Authors:** Konstantin V. Kuleshov, Sergey O. Vodop’ianov, Vladimir G. Dedkov, Mikhail L. Markelov, Andrey A. Deviatkin, Vladimir D. Kruglikov, Alexey S. Vodop’ianov, Ruslan V. Pisanov, Alexey B. Mazrukho, Svetlana V. Titova, Victor V. Maleev, German A. Shipulin

**Affiliations:** Federal Budget Institute of Science Central Research Institute for Epidemiology, Moscow, Russia (K.V. Kuleshov, V.G. Dedkov, A.A. Deviatkin, V.V. Maleev, G.A. Shipulin);; Federal Government Health Institution Rostov-on-Don Plague Control Research Institute, Rostov-on-Don, Russia (S.O. Vodop’ianov, V.D. Kruglikov, A.S. Vodop’ianov, R.V. Pisanov, A.B. Mazrukho, S.V. Titova);; Research Institute of Occupational Health, Moscow (M.L. Markelov);; Federal Budget Institute Chumakov Institute of Poliomyelitis and Viral Encephalitides, Moscow (A.A. Deviatkin)

**Keywords:** Cholera, Vibrio cholerae O1, Russian Federation, high-throughput nucleotide molecular sequence data, phylogeny, travelers, single-nucleotide polymorphism, infectious disease transmission, Haiti, Nepal, bacteria

**To the Editor:** Cholera—a severe, waterborne, virulent enteric infection caused by toxigenic strains of *Vibrio cholera*—frequently causes epidemics in developing countries and sporadic cases or local outbreaks in developed countries. The geographic features of Russia and intensive globalization have established favorable conditions for travel-associated cholera from regions to which it is endemic. During 2005–2012, six such cases occurred in Russia; these cases were related to travel from India. Three of the cases were registered in 2010, three months before the cholera outbreak in Haiti, one of the most extensive outbreaks in recent history (*1*). We genetically analyzed 4 isolates collected in 2010 and 2012 using whole-genome sequencing (Technical Appendix 1) and compared the results with a public database of representative *V. cholerae* strains (Technical Appendix 2) to identify whether these isolates were linked to cholera in Haiti and Nepal.

Isolate RND6878 was isolated on July 7, 2012 from a 28-year-old male Russian citizen. The infection was most likely caused by the patient drinking fountain water and coming into contact with river water while living in Srinagar, India. Isolate RND19191 originated from a 25-year-old female flight attendant operating a Moscow–Delhi–Moscow flight. Her infection was suspected to have occurred in Delhi during June 26–28, 2010, from ingestion of contaminated fruit. Isolate RND19187 was obtained on June 9, 2010, from a 29-year-old woman with severe cholera. Microbiological testing also confirmed the presence of *V. cholerae* in a fecal specimen from her 10-month-old daughter (isolate RND19188), even though she had no distinct symptoms of cholera. The source of infection for the woman and child was unclear but was assumed to be related to eating fruit rinsed in tap water while in the city of Vrindavan in India.

Maximum-likelihood phylogenetic analysis based on high-quality orthologous single-nucleotide polymorphisms (hqSNPs) among 75 *V. cholerae* genomes showed that all the isolates from the travel-associated cases clustered with cholera cases that occurred in 2010. The isolates from the 29-year-old woman and her daughter (RND19187 and RND19188) accurately clustered with isolates from the Nepal-3 clade (*2*) (Figure). Isolate RND19187 exhibited no hqSNP differences from VC-15 and differed from VC-18 by only 1 hqSNP. Isolate RND19191 was located in the Haiti\Nepal-4 clade and differed by only 1 hqSNP (132291G>A), located in the integrative and conjugative element encodes resistance to sulfametoxazol and trimethoprim (SXT-ICE) gene Vch1786-I0110 (Figure). RND19191 and 2010EL-1786 showed high genetic similarity and nucleotide identity to *Vibrio* pathogenic islands (VPI-1, VPI-2), *Vibrio* seventh pandemic islands (VSP-I, VSP-II), and SXT-ICE (Technical Appendix 3). Notably, isolate RND19191 has intact SXT-ICE, whereas all 3 Nepal-4 genomes have an SXT-ICE 13-gene deletion (Vch1786_I0089-I0102) (*3*). This genome also carries a *ctxB7* variant of the *ctxB* gene and five 7-mer tandem repeats (TTTTGAT). Finally, isolate RND6878 and the Haiti/Nepal-4 clade formed a well-supported monophyletic group with an estimated most recent common ancestor date of 2009 (95% CI 2008–2010) (Figure). In addition, the RND6878 genome harbored virulence-associated mobile genomic elements similar to 2010EL-1786 and contained a *ctxB7* allele and an intact SXT-ICE, but only four 7-mer tandem repeats (TTTTGAT).

**Figure:**
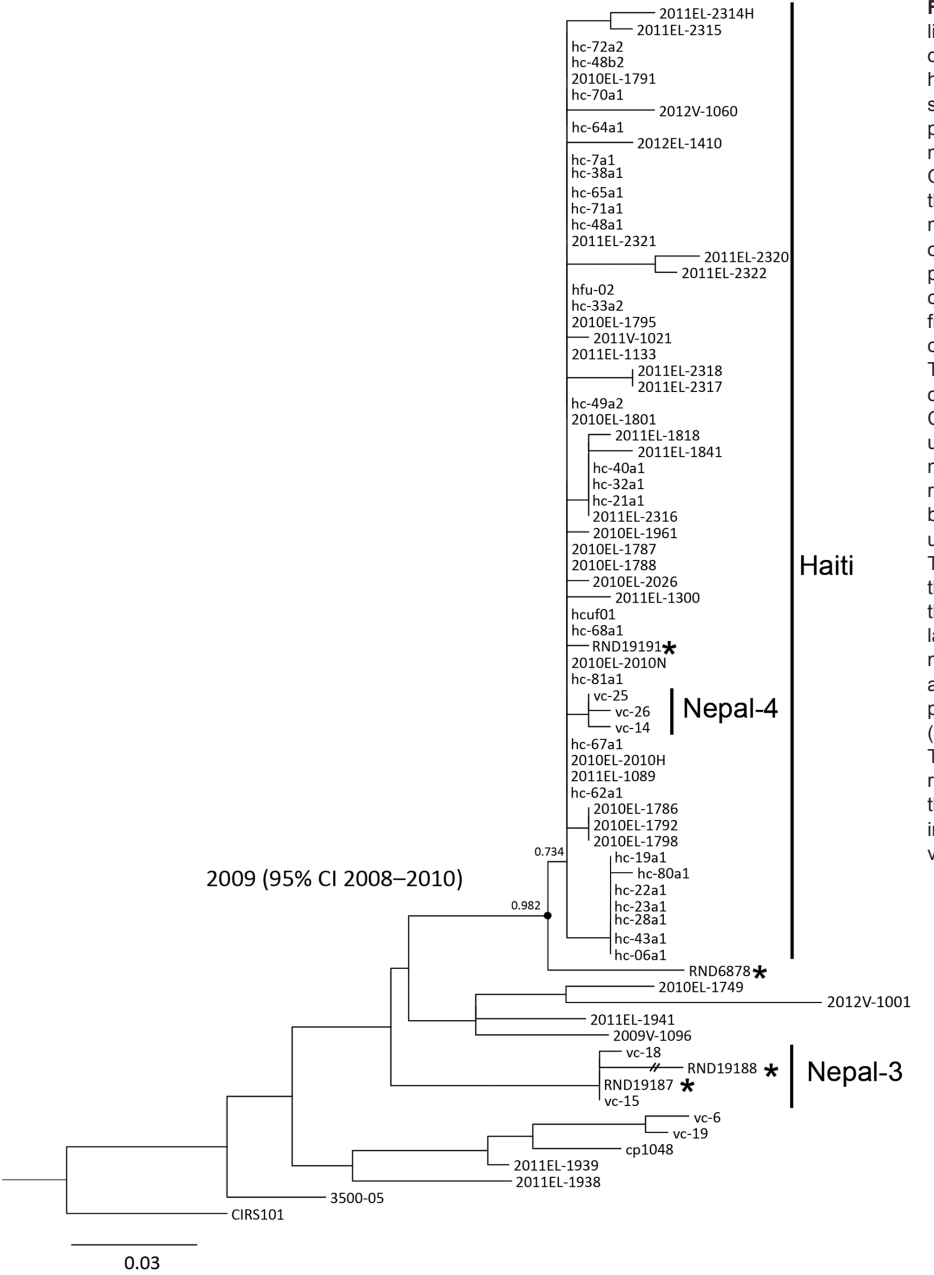
Maximum-likelihood tree based on an orthologous 193-nt–long high-quality orthologous single-nucleotide polymorphism (hqSNP) matrix of 75 *Vibrio cholerae* O1 El Tor genomes using the general time-reversible model, withestimation of invariant sites. The phylogenetic tree shows clustering of strains isolated from travel-associated cases of *V. cholerae* O1 El Tor (asterisks) with isolates collected worldwide. The CIRS101 genome was used as the outgroup. The numbers above nodes represent a statistical branch supports calculated using PhyML (black circle). The internal node between the RND6878 isolate and the Haiti/Nepal-4 clade is labeled with the estimated most recent common ancestor date, which was predicted using BEAST (http://beast.bio.ed.ac.uk). The date range provided represents the 95% CI of the estimate. Scale bar indicates substitutions per variable site.

The phylogenetic relatedness between the India and Nepal strains shows that the strains similar to the latter were first found in northern India not far from the frontier of Nepal. Collectively, these data support previously established assumptions that *V. cholerae* strains similar to those from Nepal can be detected in countries other than Nepal and Haiti (*2*). Moreover, isolate RND6878, which is phylogenetically related to the Haiti/Nepal-4 clade and was isolated in 2012, might have a common genetic lineage with the Haiti-like strains found in Nepal and northern India since 2009 (Figure). However, sequencing of representative strains isolated from different geographic regions and varying time frames is needed to reconstruct this lineage.

Remarkably, an India isolate (RND19191) from 3 months before the first cholera cases occurred in Haiti showed higher genetic similarity to the Haiti strain than Nepal isolate VC-25. This finding should be interpreted with caution because this study was limited to the analysis of only 1 isolate, with no epidemiologic context to link the isolate to the Haiti or Nepal outbreaks. Thus, India could not be validated as a primary source of Haiti strains, and the existence of a direct transmission route from India to Haiti that does not involve Nepal could not be substantiated. It is generally accepted on the basis of epidemiologic data and molecular phylogenetics that the Haiti strain was introduced from Nepal (*2*,*4*). Thus, epidemiologic studies remain critical for defining an outbreak’s origin, especially when a pathogen is rapidly disseminated by its host. This is true even when modern molecular subtyping methods, such as whole-genome sequencing, offer highly resolved phylogenetic insights.

Technical Appendix 1 Methods used for the comparative analysis of genomes in this study of travel-associated *Vibrio cholerae* O1 El Tor, Russia.

Technical Appendix 2 List of *Vibrio cholerae* O1 El Tor genomes used in this study.

Technical Appendix 3 Comparison of *Vibrio* pathogenic islands 1 and 2, *Vibrio* seventh pandemic islands I and II, and integrative and conjugative element genomic islands to the 2010EL-1786 genome based on open reading frame similarity.
