# Supplementary material for: Travel-Associated Vibrio cholerae O1 El Tor, Russia
Source: Emerg Infect Dis. 2016 Nov;22(11):2006–8. doi: 10.3201/eid2211.151727 (PMC5088018; doi:10.3201/eid2211.151727)
Supplement: Technical Appendix 1 — Methods used for the comparative analysis of genomes in this study of travel-associated Vibrio cholerae O1 El Tor, Russia. [file 15-1727-Techapp-s1.pdf]

Article DOI: http://dx.doi.org/10.3201/eid2211.151727

Travel-Associated *Vibrio cholerae* O1 El Tor, Russia

Technical Appendix 1

Methods

Identification of Single-Nucleotide Polymorphisms

To provide accurate high-quality orthologous single-nucleotide polymorphism (hqSNP) discovery and to understand the relationship between recent isolated, strains we used genome 2010EL-1786 as a reference sequence instead of generally accepted N16961. The reads obtained in this study, as well as those from NCBI Short Read Archive, were mapped to both chromosomes of reference genome 2010EL-1786 (CP003069–CP003070) by SMALT mapper (https://www.sanger.ac.uk/resources/software/smalt/) using previously described options (*1*). *V. cholerae* genomes represented as complete genomes or contigs were mapped to the reference after generating of 100 bp pseudo FASTQ reads using the "wombac-shred" Perl script (https://github.com/tseemann/wombac). Identification of single-nucleotide polymorphisms (SNPs) was performed with Freebayes v 9.9.2 (*2*). The SNPs called were filtered to remove sites with an SNP quality score <30, coverage <10, and minimum alternate fraction <75%. Additionally, we removed SNPs located in repeated regions (>90 nt long and >85% identity) of the reference genome identified by reciprocal BLASTn. SNPs located in a 10-bp window were also removed from the analysis to bypass probable mutational hot spots. As a result, a pseudoalignment of hqSNPs was used for the phylogenetic analysis with annotation by snpEff v 3.5 (*3*).

Phylogenetic Analysis

We performed a phylogenetic reconstruction of the evolutionary relationships among the seventh-epidemic relatives of *V. cholerae* using maximum-likelihood (ML). ML analyzes were performed with PhyML version 20131022 (http://www.atgc-montpellier.fr/phyml/) using the general time-reversible model with estimation of invariant sites (GTR+I). Support for the ML phylogeny was assessed by 1,000 bootstrap pseudoanalyses of the matrix data. Strains CIRS101 was used as an outgroup to root the tree. The resulting tree was visualized using FigTree v1.4.2 software (http://tree.bio.ed.ac.uk/software/figtree/).

BEAST

The analysis of the date of the most recent common ancestor with a 95% highest posterior density was based on the pseudoalignment of hqSNPs from 75 *V. cholerae* genomes. Divergence date estimates were obtained using the Bayesian Markov chain Monte Carlo framework implemented in the BEAST 2.3. software package with a reversible-jump based substitution model of nucleotide evolution (*4*). The molecular clock was calibrated using the log normal relaxed clock (*5*), which allows rates to vary on branches. The Bayesian Skyline coalescent prior (*6*) was used as a tree prior. The MCMC chain was run for 25 million generations with sampling every 2.500 generations, allowing 2.5 million generations, for burn-in to obtain an effective sample size (>200). The effective sample size was assessed using Tracer 1.5 (*7*). A maximum clade credibility tree was constructed by using Tree Annotator 2.3.0.0 and visualized with FigTree 1.4.2, which are the part of the BEAST software package.

Analysis of Virulence-Associated Regions

We implemented a simple approach to analyze similarity among the main virulence-associated mobile elements and genomic islands. Assembled contigs were directly mapped to the reference genome 2010EL-1786 (CP003069–CP003070) using Mummer v3.3 (*8*). The mapping results were converted to binary format for sequence data storage, and Samtools 0.19 (*9*) was then used to analyze particular genomic regions for the presence of insertions, deletions, and substitutions. We applied this approach to calculate both the similarity of an entire region of interest, including intergenic areas, and the similarity of coding areas related to open reading frames. In some cases, a low percentage of similarity of a given genomic region or its absence was due to the presence of these genomic regions in multiple copies throughout the reference genome. The impact of these genomic regions on similarity was not taken into account.

References

1. Katz LS, Petkau A, Beaulaurier J, Tyler S, Antonova ES, Turnsek MA, et al. Evolutionary dynamics of *Vibrio cholerae* O1 following a single-source introduction to Haiti. MBio. 2013;4:e00398–13. http://dx.doi.org/10.1128/mBio.00398-13

2. Garrison E, Gabor M. Haplotype-based variant detection from short-read sequencing. arXiv preprint arXiv:1207.3907 (2012) [cited 2016 Sep 8]. http://adsabs.harvard.edu/cgi-bin/bib_query?arXiv:1207.3907

3. Cingolani P, Platts A, Wang L, Coon M, Nguyen T, Wang L, et al. A program for annotating and predicting the effects of single nucleotide polymorphisms, SnpEff: SNPs in the genome of *Drosophila melanogaster* strain w1118; iso-2; iso-3. Fly (Austin). 2012;6:80–92. http://dx.doi.org/10.4161/fly.19695

4. Bouckaert R, Heled J, Kühnert D, Vaughan T, Wu C-H, Xie D, et al. BEAST 2: a software platform for Bayesian evolutionary analysis. PLOS Comput Biol. 2014;10:e1003537. http://dx.doi.org/10.1371/journal.pcbi.1003537

5. Drummond AJ, Ho SYW, Phillips MJ, Rambaut A. Relaxed phylogenetics and dating with confidence. PLoS Biol. 2006;4:e88. http://dx.doi.org/10.1371/journal.pbio.0040088

6. Drummond AJ, Rambaut A, Shapiro B, Pybus OG. Bayesian coalescent inference of past population dynamics from molecular sequences. Mol Biol Evol. 2005;22:1185–92. http://dx.doi.org/10.1093/molbev/msi103

7. Rambaut ASM, Xie D, Tracer AJD. v1.6 [cited 2016 Jul 1]. http://beast.bio.ed.ac.uk/Tracer. 2014.

8. Kurtz S, Phillippy A, Delcher AL, Smoot M, Shumway M, Antonescu C, et al. Versatile and open software for comparing large genomes. Genome Biol. 2004;5:R12. http://dx.doi.org/10.1186/gb-2004-5-2-r12

9. Li H, Handsaker B, Wysoker A, Fennell T, Ruan J, Homer N, et al.; 1000 Genome Project Data Processing Subgroup. The Sequence Alignment/Map format and SAMtools. Bioinformatics. 2009;25:2078–9. http://dx.doi.org/10.1093/bioinformatics/btp352
